# Low Substrate Loading Limits Methanogenesis and Leads to High Coulombic Efficiency in Bioelectrochemical Systems

**DOI:** 10.3390/microorganisms4010007

**Published:** 2016-01-05

**Authors:** Tom H. J. A. Sleutels, Sam D. Molenaar, Annemiek Ter Heijne, Cees J. N. Buisman

**Affiliations:** 1 Wetsus, European Centre of Excellence for Sustainable Water Technology, Oostergoweg 9, P.O. Box 1113, Leeuwarden 8911 MA, The Netherlands; sam.molenaar@wetsus.nl (S.D.M.); Cees.buisman@wetsus.nl (C.J.N.B.); 2 Sub-Department of Environmental Technology, Wageningen University, Bornse Weilanden 9, P.O. Box 17, Wageningen 6700 AA, The Netherlands; annemiek.terheijne@wur.nl

**Keywords:** Coulombic efficiency, BES, MET, methanogens

## Abstract

A crucial aspect for the application of bioelectrochemical systems (BESs) as a wastewater treatment technology is the efficient oxidation of complex substrates by the bioanode, which is reflected in high Coulombic efficiency (CE). To achieve high CE, it is essential to give a competitive advantage to electrogens over methanogens. Factors that affect CE in bioanodes are, amongst others, the type of wastewater, anode potential, substrate concentration and pH. In this paper, we focus on acetate as a substrate and analyze the competition between methanogens and electrogens from a thermodynamic and kinetic point of view. We reviewed experimental data from earlier studies and propose that low substrate loading in combination with a sufficiently high anode overpotential plays a key-role in achieving high CE. Low substrate loading is a proven strategy against methanogenic activity in large-scale reactors for sulfate reduction. The combination of low substrate loading with sufficiently high overpotential is essential because it results in favorable growth kinetics of electrogens compared to methanogens. To achieve high current density in combination with low substrate concentrations, it is essential to have a high specific anode surface area. New reactor designs with these features are essential for BESs to be successful in wastewater treatment in the future.

## 1. Introduction

In the past few decades, bioelectrochemical systems (BESs) have been mentioned as a promising technology for the conversion of chemical energy present in wastewaters directly into electrical energy [1], using bioanodes as the driver for different types of cathodic reduction reactions. To this point, however, BESs have not successfully been applied to the full scale to be more efficient than more conventional state-of-the-art technologies, like anaerobic digestion [2]. As we will discuss below, a crucial aspect limiting this development is the efficient extraction of electrons from complex substrates.

Microbial fuel cells (MFCs) and microbial electrolysis cells (MECs) are two specific types of BESs. Similar to all other electrochemical systems, in BESs, the reduction and oxidation reactions are spatially separated, and their occurrence can be manipulated externally by means of an electrical circuit. Essentially different from other electrochemical systems is the use of electrochemically-active microorganisms, which can exchange electrons with an electrode. In MFCs and MECs, such electrochemically-active microorganisms (also called electrogens) are used to oxidize biodegradable (organic) components from wastewater and transfer the electrons to the anode. When combined with a suitable cathode, these systems comprise a promising technology for clean and efficient recovery of energy from wastewaters [1,2,3]. When a bioanode is coupled to a cathode where a reduction reaction occurs at a higher potential than the oxidation of the substrate, for example the reduction of oxygen, energy is recovered in the form of electricity, and the system is called a microbial fuel cell (MFC) [1]. When a bioanode is coupled to a cathode where a product is formed at a potential lower than the anodic reaction, an additional electrical energy input is required, and the system is called a microbial electrolysis cell (MEC) [3,4]. The understanding of bioanodes in BESs is crucial for the application in wastewater treatment. Promising applications for the use of bioanodes are, amongst others, for the recovery of energy and nutrients from urine [5,6,7], integration in anaerobic digestion to improve methane recovery [8,9] and as the driver for different types of cathodic reduction reactions, like the production of hydrogen, but also caustic or hydrogen peroxide [10,11].

Traditionally, MFCs serve two purposes at the same time: (1) to recover renewable energy in the form of electricity; and (2) to produce clean water that can be discharged to surface waters, by removing the biodegradable material. For MFCs, the performance is often analyzed in terms of anodic current density, which reflects the specific conversion rate, and power density, which reflects the rate at which energy is recovered. Besides these performance parameters, two types of efficiencies are used to evaluate MFC performance: (1) the Coulombic efficiency (CE); and (2) the voltage efficiency (VE). The CE shows the fraction of the electrons obtained from oxidizable substrates present in the wastewater, which are recovered at the anode, indicating the efficiency of the conversion of a substrate into electrical current. When a continuous system has reached equilibrium and no change over time of both influent and effluent concentrations and measured current occurs, the CE can be calculated with:

(1) $$CE_{anode}=\frac{I_{measured}}{nF\varphi \left(C_{out}-C_{in}\right)}100\%$$

where *I_measured_* is the measured current in A, n is the moles of electrons produced per mol of substrate, F is the Faraday constant (96,485 C·mol^−1^), ϕ is the inflow rate of substrate (m^3^·s^−1^) and C_out_ and C_in_ are the substrate concentrations in the effluent and influent. Here, we only would like to consider continuously-operated systems, since for the treatment of large volumes of wastewater that are produced continuously, a reactor needs to be used that can be operated for an extended period of time.

The VE expresses the produced cell voltage (E_cell_) under load as a fraction of the equilibrium voltage at zero current (E_emf_). The latter voltage can be calculated theoretically using the Nernst equation as described in, for example, Logan *et al.* (2006) [1].

The VE is defined as:

(2) $$VE=\frac{E_{cell}}{E_{emf}}$$

**Figure 1:**
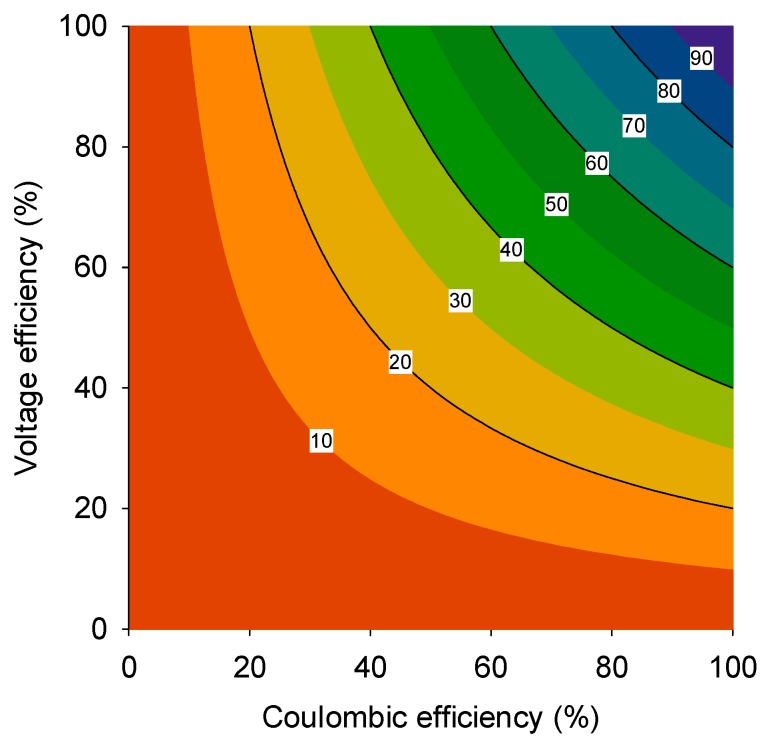
The overall efficiency of a microbial fuel cell (MFC) (contour lines in figure) plotted as a function of the voltage efficiency (%) and the Coulombic efficiency (%). To be competitive with anaerobic digestion, an overall energy efficiency of at least 30% should be achieved (adapted from [12]).

The overall energy efficiency is then calculated by multiplying the CE with the VE (Figure 1). With MFCs aiming for the production of electrical power directly, in one step from wastewater, these systems are often considered as a future alternative to anaerobic digestion (AD). AD produces methane instead of electricity and, as such, has received considerable scientific attention at the onset of BES research, because electricity is a more attractive decentral energy carrier. In view of energy recovery, an overall energy efficiency of 30% is a typical value reported to make MFCs competitive with anaerobic digestion [12]. Given the earlier definition of overall energy efficiency, it is clear that both CE and VE need to be optimized for MFCs to become an energy-efficient technology.

The optimization of VE has its boundaries: in order to obtain high VE, the observed cell voltage needs to be close to the open circuit voltage (OCV). This means, in general, that current density is low: at higher current densities, an increasing part of the cell voltage will be lost due to internal resistances, and this will decrease the VE further. Another possibility is to control the anode overpotential by control of the anode potential through a potentiostat. Generally, when the anode potential is controlled, higher current densities can be achieved than when a resistor is applied [13]. However, controlling the anode potential at higher levels goes at the expense of the harvested cell voltage and, therefore, the VE.

In general, assuming constant internal resistance, a well-designed system has 50% voltage efficiency at maximum power density. Therefore, for an MFC to be operated at maximum power density, the anode CE should be at least 60% in order to achieve an overall energetic efficiency of at least 30% (Figure 1).

It thus becomes evident that, in order to operate MFCs anywhere near their maximum power density, these systems rely on attaining appreciable anodic CEs for their overall functioning. This review summarizes the factors affecting CE, discusses how thermodynamics and kinetics affect the competition between electrogens and methanogens and gives an outlook for the prerequisites of BESs to become competitive with AD.

## 2. Main Factors Leading to CE Losses

The processes leading to losses in CE in BESs can be roughly divided into three main categories: (1) parasitic processes related to the presence of alternative electron acceptors; (2) fermentative processes; and (3) substrate consumption by biomass formation (carbon assimilation). To which extent these processes occur is determined by the Gibbs free energy and the kinetics of their underlying reactions. The consumption of substrate by biomass formation (Category 3) is imperative to the concept of a biocatalyzed electrode, as biomass needs to grow and maintain itself in order for the system to work. Thus, to some extent, CE losses due to biomass formation and maintenance are inevitable and may be kept to a minimum by a combination of operational parameters, as will be discussed later.

Regarding alternative electron acceptors (Category 1), nitrate and sulfate impose the most common threat to CE based on their frequent presence in wastewaters. With their standard reduction potentials being considerably higher than those commonly applied to anodes in BESs (Table 1), the Gibbs free energy available by substrate oxidation using these electron acceptors is higher than the Gibbs free energy for using the electrode as electron acceptor. These reactions are a property of the wastewater and can therefore not be controlled.

**Table 1:** The oxidation reduction potential range (ORP) of common compounds present in wastewater [14].

Biochemical Activity	ORP Range (mV)
Aerobic carbon oxidation	+50 to +200
Nitrification	+150 to +350
Denitrification	−50 to +50
Acidification	−40 to −200
Sulfate reduction	−50 to −250
Methanogenesis	−200 to −400

Wastewaters often contain a range of complex organic compounds, which can be fermented through a broad variety of fermentation pathways (Category 2), as reviewed by Agler *et al*. (2011) [15]. Fermentation reactions lead to the formation of shorter carbon chains out of longer ones and the formation of hydrogen and CO_2_. Most of these fermentation reactions have favorable available Gibbs free energies and/or reaction kinetics compared to anodic oxidation of complex organic compounds in BESs. When complex organic wastewaters are used as a substrate in BESs, fermentation processes will therefore dominate. On the one hand, fermentation reactions lead to VE losses, because fermentation products have higher redox potentials than their reactants, while on the other hand, fermentation reactions do not necessarily lead to CE losses, because the fermentation products, mainly acetate and hydrogen, can be oxidized at the bioanode. Pre-fermentation can even be regarded as an essential step in operating BESs fed with wastewater: so far, higher current densities in bioanodes are reached when using acetate, compared to complex organic wastewaters [16,17].

A possible disadvantage of pre-fermentation for the operation of BESs is the formation of methane. As soon as methane is formed, it can be regarded as a permanent loss to current production and, thus, CE, because it takes away the substrate for the bioanode. Table 1 gives an overview of the redox potentials of different processes occurring in wastewater treatment. As the potential of methane formation (−200 to −400 mV *vs.* Ag/AgCl) is in the same range as anode potentials commonly applied in BESs, it becomes clear that methanogenesis will be the main competing process occurring at bioanodes. The crucial question to achieve high CE in BESs is therefore: how can methanogenesis be prevented?

## 3. Substrate Loading Rate as a Control Parameter for High CE

Steering mixed cultures towards the desired product by means of process engineering is a proven strategy in many biotechnological applications. To this end, there are lessons to be learned about competition with methanogens from large-scale continuous bioreactors. An example of such a large-scale process in which the prevention of methanogenesis is crucial is biological sulfate reduction, which occurs at redox conditions similar to bioanodes.

Biological sulfate reduction is essential for the treatment of wastewaters containing sulfate, e.g., in the chemical industry. The competition between sulfate reducers and methanogens has been studied in detail; see, e.g., Visser *et al*. [18]. Monod growth kinetics of sulfate reducers on acetate are less favorable than those of methanogens. Therefore, under standard conditions, a major part of the substrate would be lost to methanogenesis. However, when operated at low substrate concentrations, sulfate reducers are at an advantage, resulting in only small amounts of substrate lost to the formation of methane. Most electrons from the electron donor will flow to sulfate reducers as long as sulfate is above 200 mg/L and no excess amount of Chemical Oxygen Demand (COD) is present (<200 mg/L). In sulfate reducing reactors, the competition is therefore solely being controlled by the substrate loading of the system.

**Figure 2:**
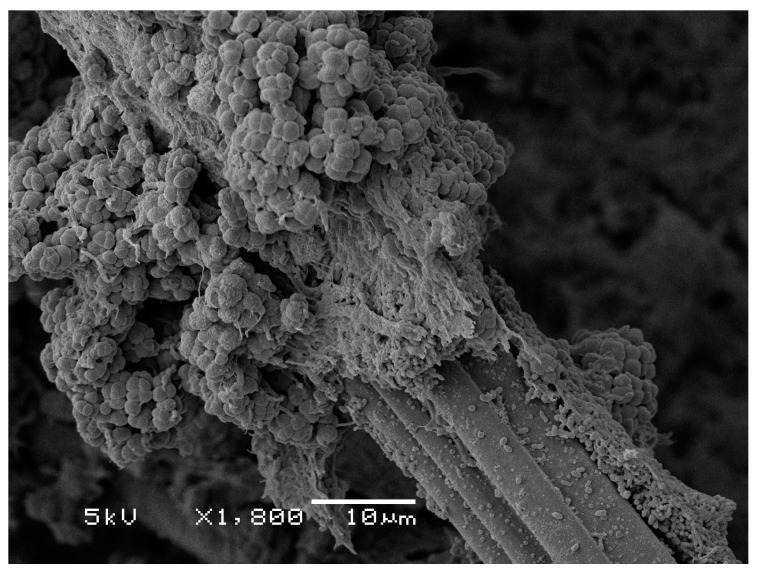
Biofilm developed on the fibers of an anode felt electrode collected from a microbial electrolysis cell [19]. An electrogenic biofilm can clearly be seen on the surface of the graphite fiber, which is covered by methanogenic microorganisms.

Electrogens, similar to sulfate reducers, use acetate as the electron donor, and therefore, similar competition strategies to keep out methanogens may be applicable for MFCs and biological sulfate reduction. Therefore, keeping substrate concentration low could be key to achieving high CE. An experimental indication of how methanogens and electrogens interact at a bio-anode is shown in Figure 2. This figure shows an anodic biofilm growing on the fiber of a graphite felt electrode while fed with acetate [19]. It can be seen that the layer of current-producing biofilm is covered by a layer of methanogens (circular cells). The outside of the biofilm is facing the bulk acetate concentration of the electrolyte and is therefore always experiencing the highest concentration of acetate available. This promotes the growth of methanogens. Obviously, the growth of a layer of methanogens on top of electrogens has a negative effect on CE, as the methanogens have the first access to the substrate. Giving electrogens a competitive advantage over methanogens from the start of operation is therefore a necessity for achieving high CE.

## 4. Anode Overpotential as a Control Parameter for High Coulombic Efficiency

Whereas sulfate reducers use sulfate as the electron acceptor, electrogens use the electrode. This provides an additional parameter to control the competition with methanogens in the case of bioanodes: the anode overpotential. For biological sulfate reduction, sulfate concentration is a rather fixed parameter depending on the type of wastewater.

The overpotential is the difference between the equilibrium potential of acetate oxidation and the actual electrode potential. The overpotential influences two processes: the rate of the reaction and the energy available for the microorganisms from the oxidation of acetate. Whereas for sulfate reduction, the energy available in the reaction is more or less constant (but dependent on SO_4_^2−^ availability), for a bioanode, the overpotential can be set in a range of potentials and, therefore, provides a way to control the Gibbs free energy available in the reaction. Next to a low substrate concentration, it provides an additional way to control the competition between microorganisms compared to conventional fermentations.

Only a few systematic studies have been performed that aim at determining the CE as a function of substrate concentration and overpotential. For example, Sleutels *et al.* (2011) showed that, to reach a CE over 80%, a substrate effluent concentration lower than 1 mM of acetate is required at an anode potential of −350 mV *vs*. Ag/AgCl or an anode potential higher than −250 mV *vs*. Ag/AgCl at an excess of acetate (>20 mM) [20]. Of course, the exact numbers highly depend on the cell design and experimental conditions, like, e.g., pH and mixing. In the following, we will discuss the fundamentals behind the competition with methanogens in more detail, by first analyzing the thermodynamics in relation to anode overpotential and, secondly, analyzing the growth kinetics of electrogens and methanogens.

First of all, the Gibbs free energy determines the energy available in the overall reaction.

Starting with acetate as the substrate, the oxidation reaction for methanogenesis, sulfate reduction and electricity production can be written as:

CH_3_COO^−^ + 4 H_2_O --> 2 HCO_3_^−^ + 9 H^+^ + 8 e^−^ (3)

For methanogenesis, the reduction reaction is:

(4) HCO_3_^−^ + 9 H^+^ + 8 e^−^ --> CH_4_ + 3 H_2_O

leading to the overall methanogenesis reaction:

CH_3_COO^−^ + H_2_O --> CH_4_ + HCO_3_^−^ (5)

The Gibbs free energy of the overall methanogenesis reaction at standard conditions is −76 kJ/mol acetate, corresponding to −9.5 kJ/mol e^−^.

For sulfate reduction, the reduction reaction is:

SO_4_^2−^ + 9 H^+^ + 8 e^−^ --> HS^−^ + 4 H_2_O (6)

leading to the overall sulfate reduction reaction:

CH_3_COO^−^ + SO_4_^2−^ --> HS^−^ + 2 HCO_3_^−^ (7)

The Gibbs free energy of the overall sulfate reduction reaction at standard conditions is −48 kJ/mol acetate, corresponding to −6 kJ/mol e^−^.

**Figure 3:**
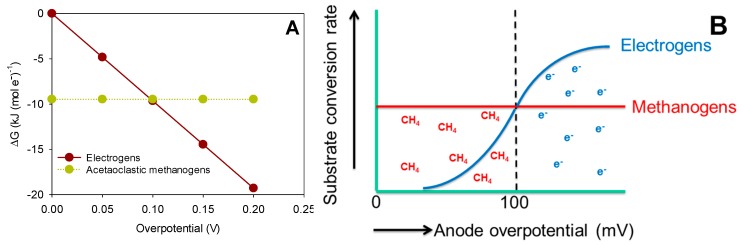
The Gibbs free energy change for acetate oxidation by electrogens is a function of the anode potential, while the growth rate of methanogens on acetate using acetate as the electron donor is independent of the anode overpotential. Consequently, at low overpotentials, methane is produced, and at higher overpotentials, current will be produced (right picture). The substrate conversion rate is related to the growth rate of the specific microorganism through the yield coefficient. Of course, the response of the specific microorganisms is dependent on other parameters, like, e.g., the type of microorganism, substrate concentration and pH.

The Gibbs free energy for methanogenesis and sulfate reduction is not dependent on the potential of the anode, but only determined by the chemical species used in the reaction. Of course, this is under the assumption that both methanogens and sulfate reducers do not interact with the anode. For the bioanode, however, the Gibbs free energy for the oxidation reaction, that uses the anode as the electron acceptor, is directly dependent on the (electric) potential of the anode, which can be controlled at any value. The Gibbs free energy for a bioanode can thus be calculated as a function of the anode overpotential, which is defined as the difference between the theoretical potential of acetate oxidation and the electric potential of the anode. The result is shown in Figure 3.

From Figure 3 it becomes clear that at an overpotential >100 mV, the electrogens can gain more energy from the oxidation of acetate than methanogens, meaning that anode potential can be used to give electrogens a competitive advantage over methanogens. This comparison is based on thermodynamic equilibrium, meaning that reaction kinetics are not taken into account.

## 5. Reaction Kinetics and Growth Parameters

When considering reaction kinetics, there are two parameters that are of importance for the competition between electrogens and methanogens: the (maximum) growth rates and the substrate affinity constants. The growth rate is important because it relates directly to the conversion rate and, thus, the current or methane production rate, via the substrate consumption rate. The growth rate μ (d^−1^) is related to the substrate consumption rate *r_s_* (g substrate-C/L^−1^·d^−1^) through:

(8) $$r_{s}=\frac{\mu X}{Y}$$

where X is the total biomass concentration (g L^−1^) and Y the yield coefficient of substrate conversion to biomass (g biomass-C/(g substrate-C)^−1^). For electrogens, the generated current is related to the substrate conversion, which is related through the yield coefficient. In turn, this yield coefficient depends on the Gibbs free energy change that is available to the electrogens [21]. The energy available for the growth of the electrogens (anabolism) is dependent on the anode overpotential; a higher overpotential leaves relatively more energy for growth and will generate more biomass per consumed amount of substrate. Consequently, the amount of electron donor lost to biomass growth will be higher at higher overpotentials. Reported values range from 0 to 0.54 g biomass-C per substrate-C used when acetate was used as the substrate [13,22,23].

Bioanode performance is normally analyzed by means of polarization curves, in which the current is measured as a function of anode potential. Because the current is related to the growth rate (assuming a relatively constant yield coefficient), a polarization curve shows the growth rate as a function of anode potential. Such a model polarization curve (see Figure 3) has a typical S-shape, as described by the Butler–Volmer–Monod model, which shows an increase in the maximum level (µ_max_) with an increase in overpotential. When overpotential is further increased, at some point, no further increase in current is achieved, because of a biochemical maximum in the conversion rate. For methanogens, the growth rate is assumed independent of anode potential. At a certain potential, the growth rate of electrogens exceeds the growth rate of methanogens, and there is a competitive advantage for electrogens.

In addition to the growth rate, also the substrate affinity constant is of importance. The effectiveness of substrate loading as a control parameter partly relies on differences in the substrate affinity constants (*K*_s_) between microorganisms, as described in the Monod model for microbial growth. The substrate affinity constant indicates at which substrate concentration half of the maximum growth rate (μ_max_/2) is reached. For electrogens, the value of *K*_s_ also depends on the anode overpotential, while methanogens are independent of the electrode potential. This effect of overpotential on the affinity constant is indicated by the slope of the polarization curves of these bioanodes. The exact relation between the overpotential and the affinity constant for substrate depends on the ability of microorganisms to use different routes to shuttle electrons through their electron transfer chain.

For electrogens, only a few studies have been performed that aimed at determining the *K*_s_ value as a function of overpotential while, for example, Lee *et al*. [24] report only a small variation of Ks as a function of anode overpotential. For methanogens, substrate affinity has been analyzed thoroughly and ranges from relatively low values of 0.5 mM for Methanotrix to relatively high values of 3.0 mM for *Methanosarcina* sp. [25]. Hamelers *et al.* [26] report two different *K*s values for two different overpotentials. They found a *K*s value of 0.35 mM at 0.1 V overpotential and of 2.2 mM at 0.2 V overpotential. This would mean that, in contrast to what was discussed before, at low overpotential, electrogens have a lower affinity constant and, thus, a competitive advantage over methanogens. In practice, however, bioanodes show higher values for CE at higher overpotentials and, therefore, do have a competitive advantage over methanogens. The analyzed values, were determined without taking biofilm growth into account and the effect of overpotential on the growth yield [27]. Clearly, there is a need for more detailed studies to determine the growth constants for mixed cultures in bioanodes and to relate these growth constants to overpotential to gain more insight into substrate concentration as a control strategy to prevent methanogenesis.

## 6. Anode Surface Area Is Key to Achieve High Rates at Low Substrate Concentrations

**Figure 4:**
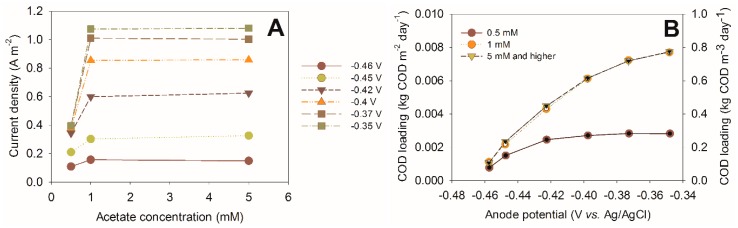
(**A**) Current density as a function of acetate concentration at different overpotentials (calculated from [28]) and (**B**) the same current density represented as COD loading as a function of the anode potential. The COD loading is expressed per m^2^ of anode surface area (left axis) and per m^3^ of reactor using a specific anode surface area of 100 m^2^·m^−3^.

Low acetate concentration is one of the key factors to keep out methanogens. This is crucial to make MFCs attractive as a competitive wastewater treatment system. Figure 4 shows an example of how acetate concentration affects the current produced by a bioanode, at different anode potentials. These experiments were performed to analyze bioanode performance with impedance spectroscopy, and the details of experimental methods can be found in [28]. At a low acetate concentration, clear differences are seen in the produced current, but once the (bulk) acetate concentration reached 1 mM, no further increase in current density was found, even at higher overpotentials.

Controlling the substrate concentrations at low levels is an attractive operational strategy for several reasons. First, it is straightforward to control by adjusting the inflow rate; second, when considering wastewater treatment, it is crucial to remove as much waste from the wastewater as possible; meaning that the outflow concentration of a biodegradable material (and thus, concentration inside the reactor) needs to be low. It is therefore inherent for application in wastewater treatment to operate the system at low substrate concentrations. To achieve a low substrate concentration, both the anode potential (influencing the current and, therefore, the COD removal rate) and the electrode surface area can be adjusted. High surface area is required, because when operating at lower substrate concentration, also the current is low, even at high overpotentials. Figure 4B shows the results of the same bioanode again, but expressed as the COD loading for the available anode surface (m^2^) and as the specific COD loading per reactor volume (calculated with a specific surface area of 100 m^2^·m^−3^). This picture shows that at overpotentials that are competitive with methanogens (<100 mV), still over 0.6 kg of COD can be removed per m^3^ of reactor per day. When a higher specific surface area would be applied in the same volume, even higher loading could be applied in the same volume, leading to higher volumetric conversion rates.

To increase this specific surface area, high surface area (porous) materials, like carbon granules, graphite brushes and graphite felt [29,30,31], are used. A problem often encountered with high surface materials is that they are often used in systems with a considerable distance between the anode and cathode, while the electrolyte has a typical limited conductivity (in the range of 1 to 5 mS·cm^−1^). For every electron that is generated, charge needs to be transported via an ionic current from the place where the electron is generated to the place where it is reduced at the cathode. A low conductivity wastewater limits the amount of ions present that can take part in this charge transport through the electrolyte and, therefore, increases the ionic resistance. For example, the ohmic voltage drop over 1 cm of electrolyte is already around 0.5 V at 10 Am^−2^ for a typical waste stream with a conductivity of 2 mS·cm^−1^ [2].

An example of a strategy to combine a high specific surface area and ionic charge transfer has been proposed in the form of a fluidized capacitive MFC [32,33,34]. In this fluidized reactor design, the anode consists of activated carbon granules that are charged and discharged in separate places. During conversion of acetate, electrons are stored in the electric double layer on the surface of the activated carbon granules. Discharge can occur in a well-designed discharge cell at locally high conductivity, thereby reducing the ohmic losses in the discharge part of the system. Further study into scalable reactor designs, in which the limitations in current by a low substrate concentration can be overcome, is important to bring bioanodes and BESs relying on bioanodes further towards application.

## 7. Conclusions

Many researchers in the field of BESs do not see a future in MFCs for the treatment of wastewater and the recovery of energy. The strategies proposed in this paper are essential for MFCs to live up to their promise and become practically applicable as a mature wastewater treatment technology. We have argued that low substrate concentration and optimized anode potential are both favorable to achieve a high CE. For MFCs to become a competitive technology in wastewater treatment, this high CE is crucial. At the same time, low substrate concentrations (effluent concentrations) need to be combined with volumetric substrate loading rates and current density; meaning that high specific surface areas are needed.
